# Horticulture producer’s willingness to participate in contract-based supply chain coordination: A case study from Queensland (Australia)

**DOI:** 10.1371/journal.pone.0285604

**Published:** 2023-05-11

**Authors:** Peggy Schrobback, John Rolfe, Delwar Akbar, Azad Rahman, Susan Kinnear, Surya Bhattarai

**Affiliations:** 1 Central Queensland University, School of Business and Law, Norman Gardens, Queensland, Australia; 2 Central Queensland University, School of Health, Medical and Applied Sciences Institute for Future Farming Systems, Norman Gardens, Queensland, Australia; Universidad Nacional Autonoma de Nicaragua Leon, NICARAGUA

## Abstract

Product export provides an option for horticulture producers in Queensland (Australia) to diversify their market and increase returns from production. Vertical supply chain coordination in the form of contract-based marketing agreements between producers and export agents/distributors could facilitate increased export. The aim of this study was to investigate the willingness of horticulture producers to participate in export focused contract-based marketing agreements. To achieve this aim, a survey including a discrete choice experiment was conducted. The results from a mixed logit model and a latent class model suggest that there are three clusters of producers: a) the export interested, b) the likely risk averse, and c) those well established in the domestic markets. Only producers in group a) expressed a preference for contract-based export marketing agreements. These producers appear to be younger, already have some export experience, and have a relatively high level of collaboration in their product supply chains. Producers in groups b) and c) expressed an interest in stronger coordination within the domestic retail sector, potentially in the form of contract farming. Prices of produce and potential higher production costs are determinants identified by all producer groups as important for their decision-making about changes to their supply chain.

## Introduction

One of the key challenges for fresh horticultural produce supply chains is the coordination of product supply among multiple stakeholders [1–3]. Such challenges include maintaining supply volumes over time, product consolidation, ensuring consistency in product quality supplied by multiple producers, and information sharing (e.g., market information, consumer demands) among various supply chain stakeholders [3]. Contractual agreements between upstream and downstream entities in fresh horticultural produce supply chains are one form of vertical coordination which can contribute to overcoming supply coordination challenges [4–7]. However, elements of contract design can affect producer’s choice about whether or not to participate in such forms of supply chain coordination [8].

The literature describes a range of studies that investigate producer’s preferences for contract-based supply chain coordination using revealed and stated preference methods [e.g., 9–15]. The main topics which the existing literature focuses on are a) contractual design attributes that affect producers’ willingness for participation, and b) socio-economic, attitudinal and motivational factors of producers that influence participation in contractual forms of supply chain coordination [e.g., 9, 10, 11, 13, 16]. Contract design specifications particularly depend on the level of economic development and institutional settings within a country (e.g., trading norms, the maturity of the legal system to enforce contracts) which is reflected in diverse types of contract attributes [e.g., 10, 13].

Within the Australian horticulture sector some contract-based forms of vertical coordination already exist between producers and downstream supply chain actors (e.g., domestic retailers). Successful hybrid forms of vertical and horizontal supply chain coordination (e.g., integration) have also been developed in recent years [e.g., 17]. However, when it comes to exporting high-quality horticulture products, it is unclear whether producers would be interested in marketing contracts with specialized export facilitators or in other mechanisms to consolidate and coordinate their product supply within the product distribution chain. This represents the research gap that this study aimed to address.

More specifically, the study investigated the willingness of Queensland horticulture producers to participate in export-focused contract-based marketing agreements with downstream buyers. The research questions were: a) What are the key attributes of formal agreements that likely encourage producer participation in vertical coordination with export focus in their fresh product supply chain?, b) What are the characteristics of producers who may seek closer vertical supply chain coordination for product export and who may not be willing to participate in such arrangements?

The Queensland horticulture industry was selected as a case study since it produces a large variety of fruits and vegetables which are at present predominantly supplied to the domestic market [18]. The industry experiences supply saturation during peak seasons which adversely affects production returns [e.g., 19]. Scope to increase horticulture exports has been identified, specifically to neighboring Asian markets [18]. To achieve the aim of this study, a survey of horticulture producers in Queensland, including a discrete choice experiment was undertaken.

The study contributes to the literature as a case study with practical implications for the Queensland horticulture industry (e.g., identification of producer groups and their need for assistance). The theoretical contribution of this research includes findings about preferred contract designs as a private solutions to supply chain coordination among producers and buyers.

The following sections include a literature review about types of agri-food supply chain coordination, materials and methods (comprising a brief overview of the Queensland horticulture industry), results, a discussion and conclusion.

## Literature review

### Vertical supply chain coordination

**Fig 1:**
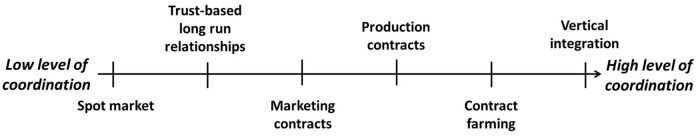
Vertical coordination continuum in agri-food supply chains. Source: Adapted from [5, 7, 26].

Vertical coordination describes how relationships between players in an agri-food supply chain are organised and is a central part of supply chain governance [4, 5, 7, 20–22]. Fig 1 offers an overview of the continuum of vertical coordination used in agri-food supply chains. Vertical coordination between supply chain actors can differ on a spectrum: at the one extreme there may be an spot market relationship where short term selling/buying interactions are coordinated by price signals only; at the other, a fully integrated relationship may occur between actors in the supply chain (e.g., production and distribution of two or more consecutive stages are undertaken under joint management and ownership) [4–7, 21, 23]. The decision about the level of vertical coordination is a strategic choice of entities within a supply chain which typically depends on their perceived benefits from the relationship relative to other options (e.g., reduced transaction costs, risk and uncertainties, increased efficiencies) and the behavioural aspects of actors (e.g., attitudes towards dependency) [5]. Furthermore, producers’ actual or perceived transactional costs (e.g., time, search, negotiation, monitoring contracts) for each of these supply chain governance options can also influence the choice of vertical supply chain coordination that is engaged in [24, 25].

### Contract-based forms of vertical coordination

Contract-based relationships between upstream and downstream entities of supply chains can be found within the far right of the continuum shown in Fig 1 [4–6, 23]. Contract-based farming is the production and supply of agri-food according to a longer-term contractual agreement between the buyer (e.g., processor, wholesaler, exporter, retailer) and the seller (producer) of a product. The contract may stipulate the conditions for the marketing, supply and production of a farm product [27]. Contracts are formal agreements between supply chain actors that are legally enforceable [23]. Such agreements can also comprise performance, behavioural and production standards [23].

Common forms of contract-based coordination between actors in an agri-food supply chain are marketing contracts, production contracts and contract farming [26]. Marketing contracts are arrangements between buyers and sellers in which the buyer agrees to provide market access and product sale for the seller’s output [7, 26, 28]. By engaging in marketing contracts the seller transfers some risks (e.g., price risk, demand risk) and the decision about where and when the product is supplied to the market to the buyer [26]. Under a marketing contract, the seller remains in control over the production process [26, 28]. This contract form differs to a production contract where the buyer of the product has more control over the production process, e.g., use of inputs and production systems or even provides the inputs and systems [7, 26, 28]. Contract farming is characterised by a high degree of coordination between producers and buyers. Under contract farming agreements, producers remain autonomous but significantly depend on centralised decision-making by the product buyer who manages the entire supply chain [7].

Engaging in forms of contract-based farming offers a range of advantages to producers, such as lowered transaction costs of selling their product, decreased market risks and uncertainties, market security, higher and more stable incomes due to higher product prices, and improved coordination with downstream buyers [11, 29, 30]. Contract-based farming is also considered to contribute to improved efficiency within a supply chain [29]. However, this form of supply chain coordination has also been criticised due to the potential loss of producers’ entrepreneurial freedom, high default rates, issues with inclusion, cheating and/or delays in payments, increase in production risks (e.g., environmental risk from monoculture cropping), and unequal bargaining power between producers and downstream actors [11, 29, 31].

From the buyer’s perspective, farming contracts offer the benefit of consistent product supply volume and varieties which confirm with quality and safety standards established within the agreement [29]. Risks associated with contract-based farming for downstream supply chain entities may include high transaction costs from contracting with many small producers, side-selling if producers decide to breach the contract and sell to others, loss in flexibility to seek alternative supply and reputational risk if things go wrong [29, 31]. Yet, contract-based forms of vertical supply chain coordination are only feasible if they prove to be beneficial to both upstream and downstream actors in a network of buyers [13, 16].

### Producer’s preference for contract design

The literature offers a range of case studies which explore producers’ preferences for contract design attributes. Most studies are from developing countries [e.g., 9–12, 32–34] presumably due to the increasing importance for contract farming in linking small-scale producers into the global value chain of agri-food products and the potential of such formal agreements to increase market access and income for producers [35]. Conversely, contract-based farming is a relatively established form of vertical coordination in developed countries [e.g., 36, 37], which may be a reason for the limited number of case studies from developed countries where the literature explores producers’ preferences towards contractual design attributes [8, 13, 38].

Except for product price which is an important attribute in all contractual studies, the literature varies significantly in the selection of contract attributes that were offered to study participants in the various case studies (e.g., payment mode, contract form, product quantity, product quality, input supply arrangements, technical assistance, data sharing, seed quality specifications, relationship to buyer and the timing of payment [9, 12, 13, 33]. This diversity in contract attributes that are used in the existing literature reflects authors’ consideration of local settings (e.g., trade norms, level of economic and institutional development) but also implies that findings from existing case studies are not necessarily comparable or transferable to other settings.

## Materials and methods

In this section, context about the Queensland horticulture industry is provided as a baseline to examine horticulture producers’ preference for closer export supply chain coordination through marketing contracts with export facilitators. This section will describe the survey design which included a discrete choice experiment to assess producers’ willingness to become involve in export-focuses marketing contracts. Data collection methods, sample statistics and data analysis methods will also be outlined.

### The Queensland horticulture industry

The Queensland horticulture industry produces a large variety of fruits and vegetables, including bananas, mangos, pineapples, avocados, tomatoes; and also nuts (e.g., macadamias, pecans, cashews) [18]. Queensland’s gross value of horticulture production is about A$3.09 billion per annum which contributes approximately 32% to Australia’s total horticulture gross production value [39]. The horticulture industry in Queensland is comprised of about 2,800 horticulture farms [40], of which many are small- or medium scale farms. The industry offers employment for approximately 25,000 people [40].

For the distribution of horticulture products to the domestic market, the wholesale supply chain model is a common approach [e.g., 41–44]. In this model, producers transfer their product to grading/pack houses which consolidate the produce from multiple producers. From the grading/pack houses the produce is forwarded to wholesalers/distribution centers or directly to local retailers. Wholesalers/distribution centers coordinate the supply of horticulture products with retailers which sell the products to consumers [e.g., 41, 43]. A variation of the wholesale model is the broker/agent model, where producers transfer the product to a broker/agent who then coordinates its distribution [44, 45]. Other distribution models that are used for the domestic market includes the direct sale approach in which producers directly supply retailers or consumers [e.g., 41, 44].

Given that only 16% of Queensland’s horticulture produce is currently exported at a total value of about A$712 million per annum, there is considerable potential to increase the export volume and value [18]. While detailed information about the current horticulture export supply chain in Queensland is limited, three types of supply chain models commonly used for fresh food exports from Australia can be identified. The first is the producer-managed export model where large producers sell and coordinate the product’s distribution process directly with importers in export markets without any other supply chain intermediaries [46]. The second is the export broker/agent model in which an intermediary coordinates the distribution from the farm to the importer in the export country, and is involved in the promotion of same, but does not actually buy the product [46]. The third is the distributor model where the product is purchased from the producer and then managed through the supply chain [46]. The latter two supply chain models are more suitable for producers that supply relatively small consignments for export.

A key limitation of the current system is that only large producers tend to be closely connected to export markets. As such, this study assesses the willingness of Queensland horticulture producers to participate in export-focused contract-based marketing agreements with downstream supply chain entities such as export agents and distributors.

### Survey and experimental design

The survey included profiling questions about the respondent’s agribusiness (e.g., type of horticulture produced, quantity produced, ownership, and current coordination levels within the supply chain) as well as the choice experiment and questions about participants’ socio-economic characteristics (e.g., age, educational attainment level). The survey questionnaire (see S1 File) and data collection methods were approved by the authors’ organizational human ethics committee (approval number: 21726).

A choice experiment is a popular method that has been used in similar studies to explore the attitudes of agri-food producers regarding farming contracts [e.g., 9–11, 13, 33, 47, 48]. As a starting point for the choice experiment participants were provided with background information about export market access and considerations for closer involvement in higher value supply chains through marketing contracts. The experiment was based on a hypothetical scenario in which participants were offered three options for their future supply chain management. These included two new supply chain options in the form of marketing contracts with exporter agents or distributors and the alternative of continuing with their current supply chain arrangement, which are largely a mix of spot pricing and trust-based relationships underpinning supply to the domestic market. Respondents were asked to choose the option which they most preferred in six repeated tasks. The three different options in each choice set were described by a consistent set of 6 attributes, each with 4 levels (Table 1).

**Table 1:** Attributes and levels of contracts in the choice experiment.

Attributes	Description	Levels
**Price increase**	Price increase refers to the increase in the product price compared to the market price that is offered by the alliance for your product.	+5% of market price +10% of market price +20% of market price +50% of market price
**Amount of produce taken**	Amount of produce taken refers to the proportion of production volume which would be supplied to the market over the length of the agreement.	10% of production per annum 20% of production per annum 50% of production per annum 90% of production per annum
**Length of agreement**	Length of the agreement refers to the time length of the contract. 0 years indicates no contract.	0 year 1 year 2 years 5 years
**Extra support**	Extra support refers to different levels of additional services offered by membership to the supply chain such as access to genetics, market and industry production information, and technological innovation.	Zero Low Medium High
**Increase in production costs**	Higher production costs refer to additional production costs due to higher control processes and specifications to meet the market requirements.	+5% +10% +20% +50%
**Increased paperwork**	Increased paperwork refers to the additional administrative tasks to meet higher protocols for market requirements.	1 day per annum 3 days per annum 7 days per annum 15 days per annum

Attribute selection was guided by the literature [e.g., 11, 13] and by consultation with representatives from Growcom, the main industry association of horticulture producers in Queensland. These attributes were assumed to best reflect the issues relevant to Queensland horticulture producers and relevant trade-offs (e.g., price increase compared with increase in production costs) and therefore may differ from attributes selected in similar studies [e.g., 13].

Product quality and traceability were not explicitly included as attributes in the experiment since it was difficult to find a consistent definition of both, across the variety of horticulture products that are produced in Queensland. To address this, participants were informed in the framing statements before the experiment that improved quality controls (e.g., standards) to meet consumer demand and increased product traceability are advantages of marketing contracts and that consideration of these aspects are the baseline for this experiment (see S1 File).

**Fig 2:**
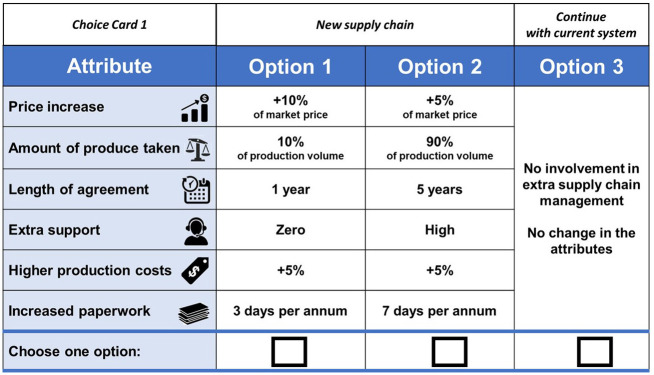
Choice task example.

The NGene software [49] was used to derive a D-efficient experimental design that minimised the D-error (0.0104) and also offered a reasonably small S-statistic (206.5). A focus group with 5 representatives from Growcom generated priors for the design. The design of 36 choice sets was blocked into six blocks of six choices sets, where participants were randomly allocated to one of the blocks. Fig 2 presents an example of one of the choice tasks that respondents were asked to choose from.

### Participant recruitment and data collection

The experiment was conducted between September 2019 and April 2020. Because it is often difficult to achieve large sample sizes with agriculture producers [e.g., 50, 51], two participant recruitment strategies were used. First, an online survey (using Qualtrics software) was conducted, which Growcom assisted by advertising and distributing the survey within their network of 2,367 members in Queensland. The second strategy was to commission a market research company to engage horticulture producers to complete the survey. A low response rate to the survey constrains the power of the subsequent analysis, but funding constraints limited the collection of a larger sample.

### Descriptive statistics

Sample statistics are shown in Table 2. Overall, 57 responses were collected, with 27 from the online survey and 30 from the market research firm. Most respondents identified as fruit producers (72%) or vegetable producers (23%), with nut and other horticultural producers accounting for 2% and 3%, respectively, in the collected sample. The high proportion of fruit producers in the sample slightly overrepresents the proportion of the total value that the Queensland fruit industry contributes to the state’s total horticulture production [18].

**Table 2:** Descriptive sample statistics.

Characteristic		N	Proportion of full sample
**Full sample**		57	100%
**Horticulture sector representation (production focus)**	Fruit	41	72%
	Vegetables	13	23%
	Nut	1	2%
	Other	2	4%
**Location in Queensland**	North (north of Bowen)	20	35%
	Central (Hervey Bay–Bowen)	13	23%
	South (south of Hervey Bay)	24	42%
**Predominant market currently supplied**	Domestic	52	91%
	Export	5	9%
**Annual income of agribusinesses (Million A$)**	0.00–1.99	35	61%
	2.00–3.99	8	14%
	4.00–5.99	5	9%
	6.00–7.99	5	9%
	8.00–9.99	0	0%
	>10.00	4	7%
**Role of the business**	Owner	20	35%
	Owner/manager	27	47%
	Family member of the owner	4	7%
	Employed manager	3	5%
	Employee	2	4%
	Other	1	2%
**Gender**	Male	43	75%
	Female	13	23%
	Prefer not to tell	1	2%
**Experience in the industry**	0–5 years	8	14%
	6–10 years	8	14%
	11–15 years	4	7%
	16–20 years	4	7%
	More than 20 years	33	58%
**Age**	18–29 years	7	12%
	30–39 years	5	9%
	40–49 years	18	32%
	50–59 years	15	26%
	60–69 years	7	12%
	70 years and older	5	9%
	Prefer not to say	0	0%
**Access to market information (more than one option could be selected)**	Agents	46	81%
	News services	14	25%
	Exporters	9	16%
	Cooperatives	10	18%
	Austrade	0	0%
	QDAF	6	11%
	Other	12	21%

Notes: ‘N’ for sample and subsample. ‘QDAF’ for Queensland Department of Agriculture and Fisheries.

The distribution of the responses by growing region shows that the central horticulture production area in Queensland is slightly underrepresented in the sample compared to the northern and southern regions.

Most (91%) of the sampled producers supply their product to the domestic market. A relatively large proportion (61%) of the surveyed farm businesses fall within the annual income bracket of A$0.50 -A$1.99 million per annum. However, there were several businesses in the sample which reported an income of more than A$2 million per annum. The dominance of small- to medium sized agribusinesses in the sample, evident as by distribution across income brackets, reflect statistics for the Australian horticulture industry [52].

Over 90% of the producers in the sample either own or own and manage their agribusiness. About 75% of the respondents were male and well over half of the sample were producers who had more than two decades of experience in the horticulture industry. The age profile shows that about 52% of the surveyed producers were 49 years of age and younger. This suggests that the age of producers in the sample was slightly lower compared to the industry average of 58 years in Queensland [53]. The respondents had a relatively high level of education with about 40% having completed either an undergraduate or postgraduate degree which aligns with statistics by ABS [54]. The sample also showed an interesting split of very experiences producer (more than 20 years experience at 56%) and relatively inexperienced producers (0–10 years at about 28%). Producers with a medium to high level of experience (11–20 years) were slightly underrepresented. The predominant sources for access to market information for participants were agents (81%), news services (25%) and cooperatives (16%).

### Choice data analysis methods

To estimate producers’ willingness to participate in export-orientated marketing contracts from the choice data, a mixed logit model (ML) was used as described by Henscher and Rose [55]. The ML model is used to estimate the probability with which producers chose one of the three supply chain options provided in the choice experiment. The model allows for random taste variations within the collected sample and permits correlations between unobserved factors over choice alternatives [55].

A latent class model is also estimated to identify different subgroups or classes among all participants in the sample who prefer the same attributes [55, 56]. Like the ML model, socio-economic characteristics are used to classify the respondents. This can provide information about potential heterogeneity among respondents.

As a final step, a Chi-square analysis was conducted to elicit additional information about latent class membership of horticulture producers. The mixed logit and latent class models were estimated with NLOGIT, an extension of the LIMDEP software.

## Results

### Mixed logit model results

The choice experiment was completed by 57 respondents, in excess of the minimum sample size identified from the experimental design (34 respondents) but below the recommended sample to generate parameter estimates significant at the 95% confidence level (207 respondents). This provided a total sample of 342 choice sets for analysis (i.e., 57 respondents x 6 completed choice cards each). To analyse the data from the choice experiment, several specifications were tested for the ML model using different possibilities for random and non-random parameters and additional variables on respondent characteristics. The model which had significant coefficients for the attributes, including the alternative specific constant (ASC), and the best model fit (e.g., highest log likelihood value, highest McFadden Pseudo R-squared and lowest Akaike Information Criteria) is shown in Table 3. The McFadden Pseudo R-squared value (0.255) fell within the range of 0.2 to 0.4, indicating a model with high explanatory power [55] (A simpler ML model without any respondent characteristics is shown in Table A.1 in S1 Appendix and demonstrates much poorer model fit statistics (e.g., log likelihood value, McFadden Pseudo R-squared)).

**Table 3:** ML model estimation results.

Parameters	Coefficient	p-value	Standard Error	95% Confidence Interval	
***Random parameters***					
**Amount of produce taken**	0.015	0.000	0.004	0.007	0.023
**Higher production costs**	-0.040	0.000	0.011	-0.061	-0.019
**Increased paperwork**	-0.034	0.162	0.024	-0.082	0.014
***Non-random parameters***					
**ASC**	-5.846	0.009	1.762	-9.299	-2.394
**Price**	0.036	0.001	0.009	0.018	0.055
**Amount of produce taken**					
**Length of agreement**	0.163	0.024	0.054	0.058	0.268
**Extra Support**	0.421	0.727	0.235	-0.039	0.881
**Higher production costs**					
**Increased paperwork**					
**Domestic Market Sales**	0.064	0.000	0.017	0.031	0.098
**Information through agent**	1.080	0.066	0.588	-0.072	2.232
**Engage with importer**	-2.768	0.002	0.914	-4.559	-0.977
**No engagement**	1.644	0.001	0.502	0.659	2.628
**Engage with retailers**	-0.942	0.071	0.522	-1.964	0.080
***Distribution of random parameters (triangular)***					
**Amount of produce taken**	0.018	0.000	0.004	0.010	0.027
**Higher production costs**	0.027	0.006	0.010	0.008	0.046
**Increased paperwork**	0.075	0.032	0.035	0.007	0.143
***Model fit statistics***					
**Log Likelihood**	-279.929				
**Log Likelihood (ASC only)**	-370.721				
**Adjusted R-squared**	0.228				
**Restricted Log Likelihood**	-375.725				
**AIC/n**	1.725				
**Chi squared**	191.593	0.000			
**McFadden Pseudo R-squared**	0.255				
***Sample***					
**Number of respondents**	57				
**Number of observations (n)**	342				

Notes: ASC for alternative specific constant, AIC for Akaike Information Criterion.

In this model, the parameters for the amount taken, higher product price and increased paperwork were treated as random. Only the coefficients for the amount taken and higher product price were significant with relatively small effect sizes suggesting the existence of heterogeneity over the sampled population around these two mean parameter estimates. However, the distributions were significant for all three random parameters, further implying that there was substantial variation in the way that participants viewed these three attributes.

The coefficients for the non-random parameters all had the expected signs and were significant. The negative sign of the ASC for the status quo option suggests that respondents tended to select Option 1 and Option 2 (new supply chains) more often relative to Option 3 (status quo). This finding implies that producers in the sample generally preferred to engage in new contract-based export supply chains over their existing supply chain system. In addition, the positive and significant coefficient for the price parameter implies that producers favour a higher product price. Producers also appear to receive a higher utility from longer agreement lengths and extra support which is indicated by the positive coefficient for both parameters. However, both effects are not very large as indicated by the small coefficient values.

It should be noted that the levels for the extra support attribute were dummy coded and that only the highest level was included in the estimation as it was the only one found to be significant. This suggests that producers only prefer a very high level of extra support in more coordinated supply chain management. Furthermore, producers’ involvement in supplying the domestic market appears to have a positive but relatively small effect in their choice for export marketing contracts. There are larger effects for producers who received market information through agents and don’t have any supply chain collaborations, confirming that those participants were more likely to choose a change option. In contrast those already engaged with importers or retailers had relatively large negative coefficients, indicating that those producers were largely uninterested in different supply options.

### Latent class model results

The identified presence of preference heterogeneity within the sample of horticulture producers for export marketing contracts (Table 3) was further explored by estimating a latent class model [55, 56]. Latent class models assume that there exists an unobservable heterogeneity in the behaviour of individuals based on which they can be categorized into discrete groups [55]. The estimation of a latent class model is useful here to derive information about the characteristics of producers who may or may not be interested in engaging in export marketing contracts.

The modelling procedure in NLOGIT searches for the optimal number of classes. For this data set the results revealed the existence of three latent classes or clusters of producers with similar preferences for the suggested supply chain management options, indicated by the significant class probabilities (see Table 4). There were similar proportions of producers in each class, at 32%, 45% and 24% for Classes 1, 2 and 3, respectively. The higher value for the McFadden Pseudo R-Square statistic and low value for the AIC/N statistic suggest a slightly stronger fitting model compared to the ML model for the same data.

**Table 4:** Latent class model estimation results.

Variable	Coefficient	p-value	Standard Error	95% Confidence Interval	
***Class 1***					
**Price**	0.247	0.003	0.083	0.085	0.410
**Amount of produce taken**	0.015	0.024	0.007	0.002	0.028
**Length of agreement**	0.463	0.001	0.143	0.182	0.743
**Extra Support**	-0.546	0.391	0.637	-1.795	0.702
**Higher production costs**	-0.254	0.003	0.084	-0.419	-0.089
**Increased paperwork**	-0.310	0.002	0.101	-0.508	-0.111
**ASC**	-21.805	0.000	6.052	-33.667	-9.943
**Experience**	-0.149	0.107	0.092	-0.329	0.032
**Age**	0.228	0.002	0.074	0.084	0.373
**Domestic Market Sales**	0.192	0.000	0.053	0.088	0.295
***Class 2***					
**Price**	0.017	0.081	0.010	-0.002	0.037
**Amount of produce taken**	0.004	0.244	0.003	-0.003	0.010
**Length of agreement**	0.118	0.036	0.056	0.008	0.228
**Extra Support**	0.736	0.006	0.268	0.211	1.261
**Higher production costs**	-0.017	0.084	0.010	-0.037	0.002
**Increased paperwork**	-0.012	0.584	0.023	-0.057	0.032
**ASC**	0.390	0.836	1.889	-3.313	4.094
**Experience**	-0.043	0.098	0.026	-0.094	0.008
**Age**	-0.016	0.556	0.027	-0.069	0.037
**Domestic Market Sales**	0.014	0.292	0.013	-0.012	0.041
***Class 3***					
**Price**	0.107	0.007	0.039	0.030	0.184
**Amount of produce taken**	0.169	0.016	0.070	0.031	0.306
**Length of agreement**	0.295	0.323	0.298	-0.290	0.880
**Extra Support**	-1.400	0.178	1.039	-3.436	0.637
**Higher production costs**	-0.131	0.007	0.049	-0.226	-0.035
**Increased paperwork**	-0.089	0.471	0.124	-0.333	0.154
**ASC**	-101.551	0.993	12272.660	-24155.520	23952.422
**Experience**	2.557	0.995	409.088	-799.241	804.355
**Age**	-0.033	0.760	0.109	-0.247	0.180
**Domestic Market Sales**	0.421	0.015	0.173	0.081	0.760
***Class probabilities***					
**Probability of Class 1**	0.314	0.000	0.072		
**Probability of Class 2**	0.448	0.000	0.070		
**Probability of Class 3**	0.238	0.000	0.064		
***Model fit statistics***					
**Log Likelihood**	-375.725				
**Log Likelihood (ASC only)**	-370.721				
**Chi-squared**	232.809	0.000			
**McFadden Pseudo R-squared**	0.310				
**AIC/n**	1.704				
***Sample***					
**Number of respondents**	57				
**Number of observations (n)**	342				

Notes: The identified three classes refer to groups or clusters of producers within the sample who exhibit similar preferences and characteristics according to their responses in the survey. ASC for alternative specific constant, AIC for Akaike Information Criterion.

The large negative ASC for Class 1 indicates that participants within this cluster were strongly in favour of a new supply chain model over the status quo system (Table 4). For those producers, higher product prices, larger amounts of produce taken, and longer agreement times were positive influences on their choices, while higher production costs and increased paperwork had negative impacts on their choices. The results for Class 1 also indicate that producers who were younger and already involved in domestic market sales were more likely to prefer new supply options.

For Class 2, the ASC was not significant which is an indication that producers in this group had no strong preference between the status quo and new supply chain options. This group preferred options with higher product prices, longer agreements and extra support, but were averse to higher production costs. However, those who had longer experience in the industry were more likely to support new options for supply chain management.

The third identified class had an ASC which was not significant suggesting, like Class 2, that producers in this cluster have limited interest in the new supply chain options (The high standard errors for the ASC and Experience attribute are likely the result of over-fitting in conjunction with the small sample size.). Members of this group prefer to engage in options that deliver higher product prices and larger amounts of produce taken and are less likely to support options with higher production costs. Producers in this cluster who were focused on domestic sales were less likely to be interested in new supply chain options.

When comparing the results across the three classes the product price and higher production costs were the only parameters which consistently influenced the choice of producers.

### Class membership analysis results

The survey collected information about participants’ self-rated business profitability compared to other businesses in the horticulture industry, their current vertical supply chain collaborations as well as their potential future collaborations (see S1 File). This data was compared with predicted membership of the three latent classes using Chi-square tests to identify additional relationships (see Table 5). Class membership was derived based on the class probability (Table 4) and participants were dummy coded to allocate them to a class. The results suggest that participants’ responses differed based on class membership for the variables: self-ranked profitability, current collaborations with retailers and consumer, current no collaboration and potential future collaborations in form of contract farming with domestic retailers. This is indicated by a statistically significant likelihood ratio for these variables in Table 5.

**Table 5:** Chi-square test results about class membership.

Variables	Class 1	Class 2	Class 3	Likelihood ratio (p-value)	Sample total (%)
**Class members (n)**	18	25	14		57 (100%)
***Self-ranked profitability compared to other businesses in the industry***					
**Less profitable**	17%	32%	0%	8.520 (0.074)	11 (19%)
**About the same profitability**	56%	44%	71%		31 (54%)
**More profitable**	28%	24%	29%		15 (26%)
***Forms of CURRENT supply chain collaborations undertaken***					
**Processors**	39%	40%	57%	1.328 (0.515)	25 (44%)
**Exporters**	39%	32%	14%	2.585 (0.275)	17 (30%)
**Retailers and consumers**	50%	28%	64%	5.307 (0.070)	25 (44%)
**Overseas importers**	11%	12%	0%	0.075 (0.963)	7 (12%)
**Contract farming with domestic retailers**	6%	4%	14%	1.362 (0.506)	4 (7%)
**No collaborations**	11%	20%	0%	4.885 (0.090)	7 (12%)
**Other collaborations**	17%	12%	7%	0.691 (0.708)	7 (12%)
***Forms of potential FUTURE supply chain collaborations***					
**Processors**	11%	8%	14%	0.381 (0.827)	6 (11%)
**Exporters**	28%	16%	36%	2.048 (0.359)	14 (25%)
**Retailers and consumers**	22%	40%	14%	3.469 (0.177)	16 (28%)
**Overseas importers**	11%	16%	7%	0.716 (0.199)	7 (12%)
**Contract farming with domestic retailers**	6%	32%	29%	6.391 (0.067)	13 (23%)
**No collaborations**	33%	28%	50%	1.900 (0.387)	20 (35%)
**Other collaborations**	6%	8%	0%	1.843 (0.098)	3 (5%)

Notes: Classes and class membership were derived in latent class analysis, see Table 4.

The self-rated business profitability of members in Class 1, who were the only cluster that preferred the export marketing contracts (Table 4), was relatively evenly distributed across the categories (less profitable, about the same profitability, more profitable) (see Table 5). This group also includes a relatively high proportion of producers who currently have some sort of vertical collaboration with other supply chain stakeholders (e.g., only 11% currently have no collaborations). Members of Class 1 include a high proportion of producers (50%) who currently collaborate with retailers and consumers (50%) but who are not predominantly interested in future contract farming arrangements with domestic retailers (6%).

The distribution of the self-rated business profitability of Class 2 members compared to the profitability of other businesses in the industry is similar to the results for Class 1. Producers in this cluster appear to have relatively few existing collaborations with retailers and consumers and limited collaborating relationships with supply chain stakeholders (i.e., 20% of group members currently have no collaborations). Among the three classes, the members of Class 2 appear to have the highest interest across all three producer clusters in exploring future contract farming options with domestic retailers (32%).

The results suggest that members of Class 3 run a relatively profitable business as they consider their operations as either about the same profitability (71%) or more profitable (29%) than other businesses in the industry (see Table 5). Furthermore, members of Class 3 appear to have established collaborations with domestic retailers and consumers (63%), and they also seem to have developed other collaborations (all members had some collaborations). Producers in the third cluster have a similar interest in future contract farming with domestic retailers (29%) as producers in Class 2.

However, it should be noted the evidence for the identified class membership characteristics is relatively weak since these relationships were only statistically significant at the 10% level.

## Discussion

The results of the study suggest that there are likely three clusters of producers present in the Queensland horticulture industry with respect to their willingness to participate in export-focused contract-based marketing agreements. The results are summarised in Table 6.

**Table 6:** Summarised results and recommendations.

Producers clusters	Class 1: The export interested	Class 2: The likely risk averse	Class 3: The domestically well established
**Characterised by**	Younger age Relatively high level of collaborations with other supply chain stakeholders Limited interest in future contract farming with domestic retailers Already involved in product export Average profitable business	Less experienced Relatively low level of collaborations with other supply chain stakeholders Interested in future contract farming with domestic retailers Average profitable business	Focus on the domestic market Very high level of collaborations with other supply chain stakeholders Interested in future contract farming with domestic retailers Average/above average profitable business
**Contract attribute conscious**	Price, amount taken, agreement length, additional costs, increased paperwork	Price, length of agreement, extra support, additional costs	Price, amount taken, additional costs
**Needs**	Facilitation of links to export agents/distributors Information about export process & export markets	Awareness of export agents/distributors Information about vertical supply chain collaboration options Information about export process & export markets Information about domestic contract farming Links to domestic retailers	Information about domestic contract farming Links to domestic retailers

The first cluster of producers can be described as ‘the export interested’ (Table 6). These producers appear keen to explore new supply chain collaborations to increase the profitability of their business given their self-rated average business profitability. They already have a high level of collaboration, experience in exporting and are not specifically interested in future domestic contract farming. Not surprisingly, this group of producers appears to consider most of the proposed contract attributes in their response to the choice tasks (Table 4). Ways to support these producers in engaging with export agents/distributors may include the provision of links between the stakeholders, general information about export processes and export market information. This support could be provided to producers by horticulture industry associations such as Growcom but also by agricultural extension staff of the Queensland Government.

The second cluster of producers within the Queensland horticulture industry can be described as ‘the likely risk averse’ regarding their willingness to participate in export marketing agreements (Table 6). Their low level of collaboration with other supply chain stakeholders, their interest in future contract farming with domestic retailers, their self-rated average business profitability, and their low level of experience as horticulture producers suggests that they may prefer to maintain their current supply chain collaborations as less risky product distribution options. Other potential reasons for the hesitation of producers in this group to become engaged in export marketing contracts and improved supply chain coordination may include lack of trust, attitude, competence as entrepreneurs, networks embeddedness, and negative experiences with collaborations [e.g., 8, 30] or associated actual or perceived transaction costs [e.g., 24, 25]. Additional research is needed to verify potential barriers for the willingness of members of this cluster to participate in export-focused marketing contracts.

Given the interest of producers in Class 2 in future contract farming with domestic producers, horticulture industry associations and/or government extension staff should facilitate the distribution of information about domestic contract farming options (e.g., their advantages and disadvantages), and facilitate links between the respective supply chain stakeholders. Furthermore, information about options of vertical supply chain collaboration (Fig 1) may assist this producer cohort in broadening their network coordination alternatives.

The third cluster of horticulture producers can be described as ‘the domestically well established’ (Table 6). These producers show a clear preference in supplying the domestic market, have a very high level of collaboration, rate the profitability of their business as average or above average, and are interested in future contract farming with domestic retailers. These are potential reasons for their lack of interest in export marketing contracts. Interestingly, members of Class 3 appear to have only considered the attributes price, amount taken and costs in their choice which could be another indication for their lack of interest export marketing contracts. Hence, the provision of information about agents/distributors, export process and export markets appear to be less important for this group due to the limited interest in product export. As for producers in Class 2, information about contract farming with domestic retailers and facilitation of links to domestic retailers may be beneficial for members of Class 3.

Although the combination of contract attributes that were considered as relevant in the decision about export marketing contracts varies across the three classes of producers, product price and additional production cost appear to be the key attributes that all producers regard as important. These findings may not be surprising as both attributes determine production profitability which is typically one of the key organisational goals for agribusinesses [57, 58]. Previous studies that investigated producers’ willingness to participate in contact-based supply chain collaboration agreements reported similar results for the price attribute [e.g., 10, 13, 15]. Yet, additional costs as a key attribute for contractual agreements between agri-food producers and downstream stakeholders (e.g., export agents, retailers) have not been considered in the extant literature [e.g., 10, 13, 15]. This emphasizes the importance of the individual context (e.g., economic development, institutional setting, trading norms) to be considered in designing vertical supply chain coordination agreements.

While, ‘the export interested’ producer cluster seem to consider most of the proposed contract attributes, it is likely that the design of export marketing contracts needs to be negotiated individually with producers rather than offering all interested agribusinesses a uniform contract. Alternatively, a contract that offers a basic framework but provides flexibility within the terms for producers that best suits their operations and preferences may be considered. This is important to ensure that producers’ interest in contractual agreements is met and that their needs are not being bypassed [8, 13]. Since contracts are private solutions for organising the supply chains, they can only be viable if there is a benefit for both producers and buyer [13].

The composition of the identified three producer classes differ from the producer clusters identified in previous studies [9, 10, 13, 59]. This is likely due to the experimental design (e.g., use of different attributes in choice experiments), the application to different agriculture industries (e.g., beef, horticulture, hog, coffee), type of contractual supply chain coordination (e.g., marketing contract, contract farming) and potentially also to cultural aspects that affect decision making in different countries [10, 13, 30, 59].

A major limitation of the study is the small sample size, which is a common constraint when surveying the agriculture sector [e.g., 51, 60]. While the sample size was 67% above the minimum level specified in the experimental design (i.e., 34), it was still well below the recommended size (i.e., 207). It is also important to note that there was some variation in the priors used to generate the experimental design from an industry workshop and the final results, reflecting the limited information available to generate efficient designs in this topic area. The review by de Bekker-Grob et al. [61] identified that about one third of discrete choice experiments had sample sizes less than 100, noting that small size effects may still be meaningful, but that larger samples are typically required to detect small effects. For this study, the rule of thumb proposed by Orme [62] and Johnson and Orme [63] is applied: N > 500c / (t x a), where N is the required sample size, *c* is the largest number of levels in the design (4) (see Table 2), *t* equals the number of choice tasks (6) and a equals the number of alternatives (3) (see Fig 2). The resulting sample size of 111 is almost double what was achieved in the present study and confirms that while results are still meaningful, they have a limited ability to identify small effects.

Furthermore, a potential bias could be the design of the choice card (Fig 2). Option 3, the status quo, is visually different to the other two options, i.e., text phrase taking up the entire column, which could have led to a selection bias. Hence, the results of this study should be considered cautiously, and additional assessments should be undertaken to verify the robustness of the findings.

## Conclusion

The aim of this study was to investigate the willingness of Queensland horticulture producers to participate in export-focused marketing agreements with export agents/distributors as a form of closer vertical supply chain coordination. The findings suggest that there is one producer cohort within the industry that is eager to participate in such agreements. Two other producer cohorts appear to have limited interest in engaging in export marketing agreements. Contract attributes that were identified as important to all participants in their decision about engaging in export marketing agreements included product price and potential increased costs of production. The results also identified that producers who have no preference for exploring export marketing agreements, were interested in closer coordination with the domestic retail sector such as through contract-based farming.

However, caveats relating to the relatively small sample size used for analysis in this study were identified and discussed. Therefore, the findings should be treated cautiously and regarded as a pilot study. Future research could assess export barriers as perceived by horticulture producers in more detail (e.g., identification of export barrier categories, comparison of export barriers with other agriculture sectors). This could provide additional information why some producers cohorts who may benefit economically (e.g., Class 2) have a limited willingness to participate in export-marketing contracts or in product export more generally.

A theoretical contribution of this case study is the identification producer’s motivation for participation in (context-specific) marketing contracts for the export of horticulture products. From a practical perspective, the study offers information about producer’s preferences for closer coordination within their supply chains as well as specific contract attributes and potential trade-offs between these attributes which may underpin their participation. The findings can be useful to develop strategies to achieve stable producer-buyer relationships and to increase the participation of horticulture producers in global agri-food supply chains which subsequently can contribute to regional economic growth.

## Supporting information

S1 File (DOCX)

S1 Appendix (DOCX)
