# Supplementary material for: Horticulture producer’s willingness to participate in contract-based supply chain coordination: A case study from Queensland (Australia)
Source: PLoS One. 2023 May 11;18(5):e0285604. doi: 10.1371/journal.pone.0285604 (PMC10174511; doi:10.1371/journal.pone.0285604)
Supplement: S1 File — (DOCX) [file pone.0285604.s001.docx]

**Supplementary material**

**Horticulture producer’s willingness to participate in contract-based supply chain coordination:**

**A case study from Queensland (Australia)**

**Peggy Schrobback, John Rolfe, Delwar Akbar, Azad Rahman, Susan Kinnear, Surya Bhattarai**

**Corresponding author:** Peggy Schrobback, [peggy.schrobback@csiro.au](mailto:peggy.schrobback@csiro.au)

**SURVEY QUESTIONNAIRE**

**PART A: Questions about your business**

**Q1: Which is the main horticulture industry that your business belongs to?**

|  | Mango | |
| --- | --- | --- |
|  |  | |
|  | Lychee | |
|  |  | |
|  | Avocado | |
|  |  | |
|  | Other, please specify: __________________________________ | |
|  |  |  |
|  |  |  |

**Q2: How many people are on average employed in your agri-business? Please enter:**

|  | Full time | Part time | Casual (Harvest period) | Casual (Rest of the year) |
| --- | --- | --- | --- | --- |
| 1. Family members |  |  |  |  |
|  |  |  |  |  |
| 1. Employees |  |  |  |  |

**Q3: What is the approximate annual production volume of your agri-business? Please enter answer in applicable unit field:**

|  | Tonnes **OR** |
| --- | --- |
|  |  |
|  | Boxes/Trays |

**Q4: What is the average price for the selected unit of your product that you received in the past year? Please enter answer in the same applicable unit field:**

|  | $/Tonnes **OR** |
| --- | --- |
|  |  |
|  | $/Box/Tray |

**Q5: What proportion of your total crop is your business currently supplying to the following markets? Please enter (the total needs to add up to 100%):**

|  | | % Domestic | |
| --- | --- | --- | --- |
|  | |  | |
|  | | % Export | |
|  |  | |  |

**Q6: How would you rate the profitability of your agri-business compared to other businesses in your industry? Please circle the relevant option:**

|  | Much less profitable | Less profitable | About the same profitability | More profitable | Much more profitable |
| --- | --- | --- | --- | --- | --- |
| Profitability of your business compared to other local businesses in the industry | 1 | 2 | 3 | 4 | 5 |

**Q7: Please enter the post code where your main agri-business is located:**

|  |  |
| --- | --- |

**Q8: What is the ownership structure of your agri-business?**

|  | Family owned |
| --- | --- |
|  |  |
|  | Mix of family owned and commercial entities |
|  |  |
|  | Corporate |
|  |  |
|  | Other, please specify:___________________________________________________ |

**Q9: Is your business a member of industry organisations? Please select all options that apply:**

|  | Growcom |
| --- | --- |
|  |  |
|  | Hort Innovation |
|  |  |
|  | Local cooperative, please specify which: ___________________________________ |
|  |  |
|  | None |
|  |  |
|  | Other, please specify:__________________________________________________ |

**Q10: Where do you source market information to determine options to supply your product to the market?**

|  | Agents |
| --- | --- |
|  |  |
|  | News services |
|  |  |
|  | Exporters |
|  |  |
|  | Cooperatives |
|  |  |
|  | Austrade |
|  |  |
|  | Other, please specify: __________________________________________________ |

**Q11: Supply chain collaboration refers to interactions between businesses and organisations for the purposes of improving market access through to consumers.**

**Which form of supply chain collaboration is CURRENTLY the MOST IMPORTANT for your agri-business? Select the relevant option:**

|  | Spot market |
| --- | --- |
|  |  |
|  | Trust-based relationships |
|  |  |
|  | Farming contracts (e.g., marketing or production contract) |
|  |  |
|  | Joint venture |
|  |  |
|  | None of these |

***[Instructions for Q11 programming:***

*The following explanation should pop-up when respondents hold their cursor over “Spot market”:*

***Spot market*** *relationships are short-term contracts which are coordinated by demand and supply in the market.*

*The following explanation should pop-up when respondents hold their cursor on “Trust-based relationships”*

***Trust-based relationships*** *refers to relationships which are not bound by contracts but build on interpersonal and/or long-standing connections.*

*The following explanation should pop-up when respondents hold their cursor on “Farming contracts …)”:*

***Farming contracts*** *refer to formal selling and buying agreements. These agreements may differ depending on the contract type (e.g., marketing contract, production contract).*

*The following explanation should pop-up when respondents hold their cursor on “Joint venture”:*

***Joint venture*** *refers to a relationship where farmers and other actors of the supply chain have joint ownership of resources (e.g., farmland, processing equipment).]*

***Q11 (2nd part): Depending on the answer selected for Q11 (see above) the following options should be displayed:***

**Q11a: Please select the supply chain players that you consider as having an important spot market-based relationship with:**

|  | Agents |
| --- | --- |
|  |  |
|  | Processors |
|  |  |
|  | Wholesalers |
|  |  |
|  | Exporters |
|  |  |
|  | Retailers |
|  |  |
|  | Consumers |
|  |  |
|  |  |
|  | None of these |

**Q11a: Please select the supply chain players that you consider as having an important trust-based relationship with:**

|  | Agents |
| --- | --- |
|  |  |
|  | Processors |
|  |  |
|  | Wholesalers |
|  |  |
|  | Exporters |
|  |  |
|  | Retailers |
|  |  |
|  | Consumers |
|  |  |
|  | Other farmers |
|  |  |
|  | None of these |

**Q11a: Please select the supply chain players that you consider as having an important contract-based relationship with:**

|  | Agents |
| --- | --- |
|  |  |
|  | Processors |
|  |  |
|  | Wholesalers |
|  |  |
|  | Exporters |
|  |  |
|  | Retailers |
|  |  |
|  | Consumers |
|  |  |
|  | None of these |

**Q11a: Please select the supply chain players that you consider as having an important joint venture-based relationship with:**

|  | Agents |
| --- | --- |
|  |  |
|  | Processors |
|  |  |
|  | Wholesalers |
|  |  |
|  | Exporters |
|  |  |
|  | Retailers |
|  |  |
|  | Other farmers |
|  |  |
|  | None of these |

**Q12: Which EXTRA form(s) of supply chain collaboration would your agri-business potentially LIKE TO ENGAGE WITH IN FUTURE? Select all relevant options:**

|  | Close relationships with processors |
| --- | --- |
|  |  |
|  | Collaboration with exporters |
|  |  |
|  | Close relationships with retailers and consumers |
|  |  |
|  | Collaboration with overseas importers |
|  |  |
|  | Contract farming for the retail sector (e.g., Woolworths, Coles, Aldi) |
|  |  |
|  | Informal information sharing agreements with other producers |
|  |  |
|  | Collaboration with other producers on the production side (e.g., technical assistance) |
|  |  |
|  | Collaboration with other producers on product marketing aspects (e.g., product promotion, market demand analysis) |
|  |  |
|  | None |
|  |  |
|  | Other, please specify: _______________________________________ |

**Q13: What do you consider as barriers for more collaboration within the supply chain of your product? Please rank these barriers from 1 = LARGEST PERCEIVED BARRIER to 9 = LEAST PERCIVED BARRIER:**

|  | Losing production/supply flexibility and independence in supplying different markets | |
| --- | --- | --- |
|  |  | |
|  | Finding producers who would like to form an alliance | |
|  |  | |
|  | Sharing commercially sensitive information | |
|  |  |  |
|  | Lack of trust | |
|  |  | |
|  | Costs to meet higher market specifications | |
|  |  | |
|  | Lack of leadership | |
|  |  | |
|  | Lack of knowledge about other stage of the supply chain | |
|  |  | |
|  | Lack of external support (e.g., government, industry organisations) | |
|  |  | |
|  | Lack of time and commitment to explore closer collaborations supply chain | |
|  |  | |
|  | Other, please specify: _______________________________________ | |

**PART B: Choice Experiment**

**Market Access Context**

Improved market access can generate higher returns for agricultural products. Limited access to domestic and export markets has been identified as one of the major barriers for the horticulture industry in Queensland to achieve a higher product value.

There have already been steps taken to improve market access through:

- Free trade agreements with overseas countries
- Improvements in transport links
- Government incentives to support market access

**However, more could be done.** Supply chain management actions that are likely to result in higher product value include:

- Closer links between producers and other stages of the supply chain (e.g., processors, wholesalers, and exporters),
- Improved quality controls to meet consumer demand,
- Increased traceability and feedback from consumers.

This survey investigates horticulture producer’s interest in achieving higher product returns by participating in improved supply chains. The research question to be investigated is:

**What is the extent of producer interest in higher value supply chains?**

**To better understand this relationship, we need your participation.**

**Considerations**

As part of this survey we would like you to make some choices about potential supply chain management options.

On the following pages you will be shown different scenarios which offer options for close involvement in higher value supply chains for your product.

Most of the options would improve access to higher value markets but would involve changes to your supply chain management, cost and control.

We want to identify if you would be interested in joining supply chains that would offer higher prices for some of your product; but would also involve higher standards for quality assurance with higher costs and more paperwork to meet these standards.

**When making your choices please consider that**:

- Involvement in higher value supply chains has both costs and benefits,
- We are just presenting the most relevant factors that might be involved,
- There may be other important issues for your farm operation,
- Each farming operation is different, and there are no right or wrong answers,
- The scenarios are hypothetical, but are based on current knowledge about what could happen,
- Please make your choices as if they were real.

**Choice Tasks**

This experiment involves six choice tasks about potential involvement in a new market for your crop each year. The choices for each task may look very similar, but they do differ. **Please treat each page separately.**

*[Instructions for explanatory choice card programming: Explanatory box for each attribute to pop-up when respondents hold their cursor over respective attribute name. Explanatory box for attribute level to pop-up when respondents hold their cursor over respective box frame on choice card.]*

**CHOICE CARD EXAMPLE**

***Price increase*** *refers to the increase in the product price compared to the market price that is offered for your product by the agreement.*

***Length of agreement*** *refers to the time length of the contract.0 years indicates no contract.*

***Attribute levels*** ***for International and Domestic market options*** *will change for each Choice Card.*

***Amount of produce taken*** *refers to how selective the new buyers will be – the lower the proportion the tighter the standards and selection.*

**
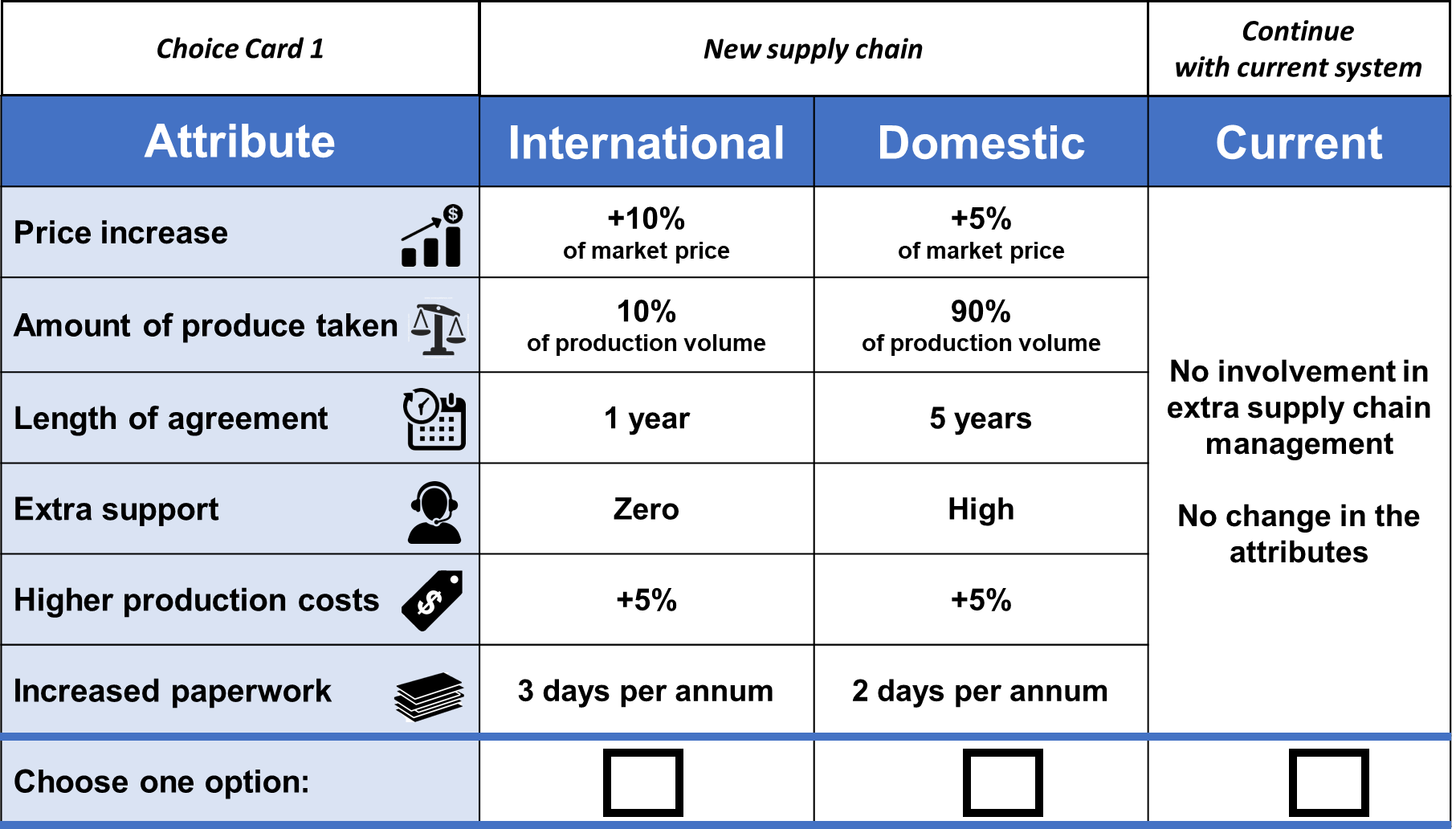
**

***Attribute levels for the Current market option*** *will remain the same for each Choice Card. Selecting this option means no extra involvement in supply chain management would be preferred, or you are unsure about the options.*

***Increased paperwork*** *refers to the additional administrative tasks to meet higher protocols for market requirements.*

***Extra support*** *refers to different levels of additional services offered by membership to the supply chain such as access to genetics, market and industry production information, and technological innovation.*

***Higher production* costs** refers to additional production costs due to higher control processes and specifications to meet the market requirements.

**CHOICE CARD 1-6 to follow (here example only)**

Please select the option that you most prefer based on your experiences in your agri-business.

**
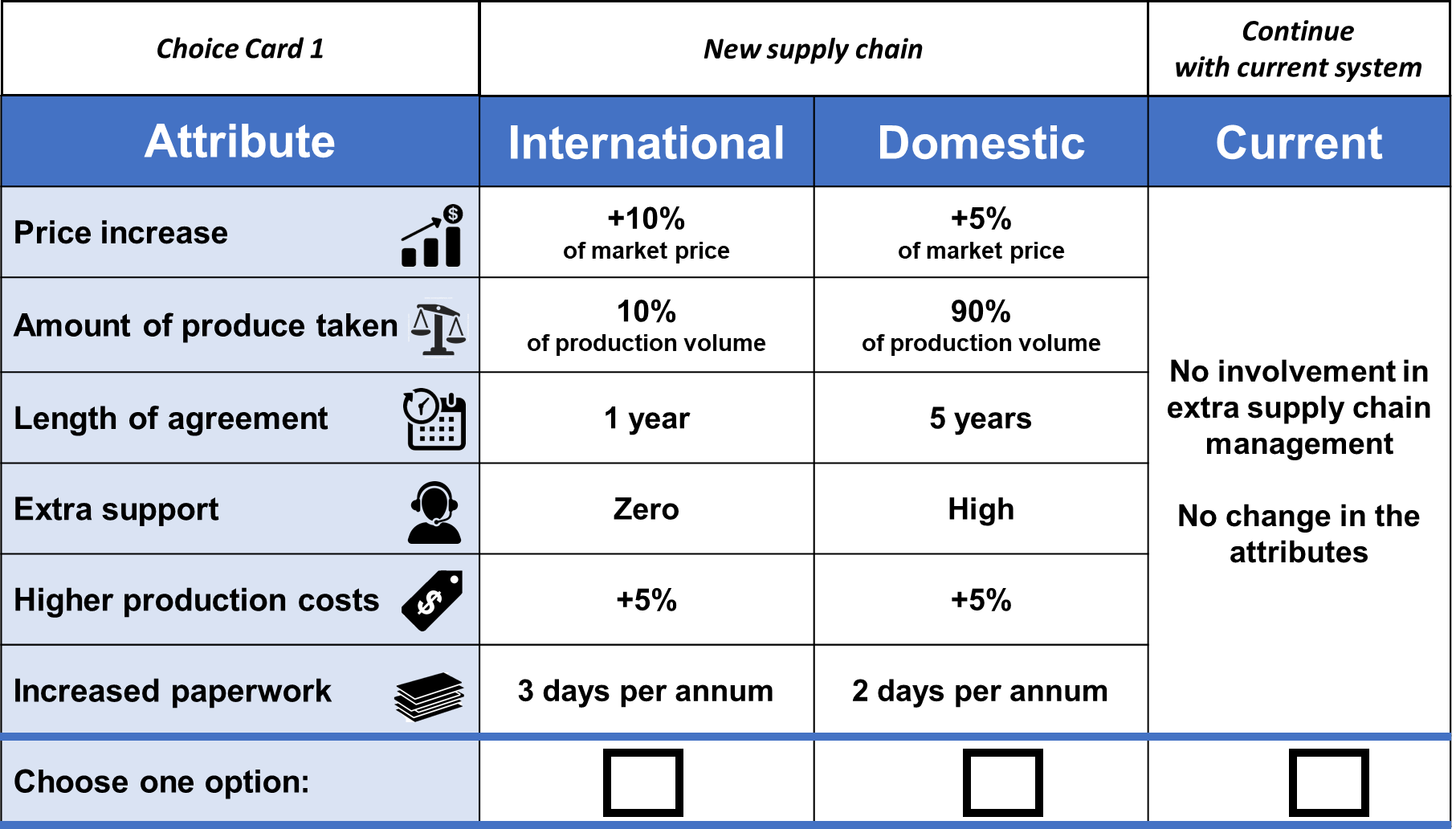
**

**Your experience in completing the choice tasks**

**Q20. For the choices you have just made, please score the following statements from (1) STRONGLY AGREE to (5) STRONGLY DISAGREE. Circle the relevant number.**

|  | Strongly agree | Agree | Do not know | Disagree | Strongly disagree |
| --- | --- | --- | --- | --- | --- |
| 1. I am confident that I made the correct choices. | 1 | 2 | 3 | 4 | 5 |
|  |  |  |  |  |  |
| 1. I understood the information in the questionnaire. | 1 | 2 | 3 | 4 | 5 |
|  |  |  |  |  |  |
| 1. I needed more information than was provided. | 1 | 2 | 3 | 4 | 5 |
|  |  |  |  |  |  |
| 1. I found the choice options to be credible. | 1 | 2 | 3 | 4 | 5 |
|  |  |  |  |  |  |
| 1. I found the choice options confusing. | 1 | 2 | 3 | 4 | 5 |
|  |  |  |  |  |  |

**Q21. When answering each of the choice situations, did you ALWAYS choose the “No involvement in extra management” option? If yes, which of the following most closely represents your reasons? Tick one box only.**

|  | Making supply chain improvements is not important. |
| --- | --- |
|  |  |
|  |  |
|  | I support supply chain improvements but do not like to collaborate with others. |
|  |  |
|  | I support supply chain improvements but worry about costs involved. |
|  |  |
|  | I found the choice options confusing. |
|  |  |
|  | I prefer my current option to continue as it is. |
|  |  |
|  | Other reason. Please specify: _________________________________________ |

**PART C: Questions about you**

**Q22: Which position best describes your role in the agri-business?**

|  | Owner |
| --- | --- |
|  |  |
|  | Owner/Manager |
|  |  |
|  | Family member of the owner |
|  |  |
|  | Employed manager |
|  |  |
|  | Employee |
|  |  |
|  | Other, please specify:__________________________________________ |

**Q23. What is your gender?**

|  | Male |  | Female |  | Prefer not to tell |
| --- | --- | --- | --- | --- | --- |

**Q24: How long have you been working in the industry?**

|  | 0-5 years |
| --- | --- |
|  |  |
|  | 6-10 years |
|  |  |
|  | 11-15 years |
|  |  |
|  | 16-20 years |
|  |  |
|  | More than 20 years |
|  |  |

**Q25. How old are you?**

|  | 18-29 |
| --- | --- |
|  |  |
|  | 30-39 |
|  |  |
|  | 40-49 |
|  |  |
|  | 50-59 |
|  |  |
|  | 60-65 |
|  |  |
|  | 66 and older |
|  |  |
|  | Prefer not to say |
|  |  |

**Q26. What is the highest level of education you have obtained?**

|  | Primary school |
| --- | --- |
|  |  |
|  | High school |
|  |  |
|  | Post school qualification (TAFE/Trade certificate) |
|  |  |
|  | Undergraduate degree |
|  |  |
|  | Postgraduate degree |
|  |  |

**Q27. Please indicate the total weekly income (before taxes) that you and your partner (if applicable) currently earn.**

|  | less than $499 per week ($25,999 per year) |
| --- | --- |
|  |  |
|  | $500 – $799 per week ($26,000 – $41,599 per year) |
|  |  |
|  | $800 – $1,199 per week ($41,600 – $62,399 per year) |
|  |  |
|  | $1,200 – $1,699 per week ($62,400 – $88,399 per year) |
|  |  |
|  | $1,700 – $1,999 per week ($88,400 – $103,999 per year) |
|  |  |
|  | $2,000 – $2,999 per week ($104,000 – $155,999 per year) |
|  |  |
|  | $3,000 or more per week ($156,000 per year) |
|  |  |
|  | Prefer not to say |

**Q28. What is the proportion of your household income from your employment in the agri-business?**

|  | 0-24% of annual net income |
| --- | --- |
|  |  |
|  | 25-49% of annual net income |
|  |  |
|  | 50-74% of annual net income |
|  |  |
|  | 75-100% of annual net income |
|  |  |

**You have fully completed this survey!**

**Thank you very much for your time.**
