# Supplementary material for: Horticulture producer’s willingness to participate in contract-based supply chain coordination: A case study from Queensland (Australia)
Source: PLoS One. 2023 May 11;18(5):e0285604. doi: 10.1371/journal.pone.0285604 (PMC10174511; doi:10.1371/journal.pone.0285604)
Supplement: S1 Appendix — (DOCX) [file pone.0285604.s002.docx]

**Appendix**

**Table A.1. Attributes only ML model estimation results.**

| **Parameters** | **Coefficient** | **p-value** | **Standard Error** | **95% Confidence Interval** | |
| --- | --- | --- | --- | --- | --- |
| ***Random parameters*** |  |  |  |  |  |
| **Amount of produce taken** | 0.007 | 0.060 | 0.004 | 0.000 | 0.015 |
| **Higher production costs** | -0.058 | 0.000 | 0.014 | -0.085 | -0.030 |
| ***Non-random parameters*** |  |  |  |  |  |
| **ASC** | 0.781 | 0.008 | 0.294 | 0.204 | 1.358 |
| **Price** | 0.035 | 0.000 | 0.009 | 0.017 | 0.052 |
| **Length of agreement** | 0.145 | 0.003 | 0.049 | 0.048 | 0.241 |
| **Extra Support** | 0.366 | 0.097 | 0.220 | -0.066 | 0.797 |
| **Increased paperwork** | -0.025 | 0.193 | 0.019 | -0.061 | 0.012 |
| ***Distribution of random parameters (triangular)*** | | | | | |
| **Amount of produce taken** | 0.020 | 0.000 | 0.005 | 0.010 | 0.030 |
| **Higher production costs** | 0.050 | 0.000 | 0.014 | 0.023 | 0.078 |
| ***Model fit statistics*** |  |  |  |  |  |
| **Log Likelihood** | -311.195 |  |  |  |  |
| **Log Likelihood (ASC only)** | -370.721 |  |  |  |  |
| **Adjusted R-squared** | 0.171 |  |  |  |  |
| **Restricted Log Likelihood** | -375.725 |  |  |  |  |
| **AIC/n** | 1.872 |  |  |  |  |
| **Chi squared** | 129.06 | 0.000 |  |  |  |
| **McFadden Pseudo R-squared** | 0.172 |  |  |  |  |
| ***Sample*** |  |  |  |  |  |
| **Number of respondents** | 57 |  |  |  |  |
| **Number of observations (n)** | 342 |  |  |  |  |

ASC for alternative specific constant, AIC for Akaike Information Criterion.
